# Oral Delivery of Nucleic Acids with Passive and Active Targeting to the Intestinal Tissue Using Polymer-Based Nanocarriers

**DOI:** 10.3390/pharmaceutics13071075

**Published:** 2021-07-13

**Authors:** Sagun Poudel, Prabhat R. Napit, Karen P. Briski, George Mattheolabakis

**Affiliations:** School of Basic Pharmaceutical and Toxicological Sciences, College of Pharmacy, University of Louisiana Monroe, 1800 Bienville Dr., Monroe, LA 71201, USA; poudelsa@warhawks.ulm.edu (S.P.); napitpr@warhawks.ulm.edu (P.R.N.); briski@ulm.edu (K.P.B.)

**Keywords:** mannosylated PEI, nanoparticles, oral delivery, nucleic acids, targeting

## Abstract

Despite the apparent advantages for long-term treatment and local therapies against intestinal diseases, the oral delivery of nucleic acids has been challenging due to unfavorable physiological conditions for their stability. In this study, a novel nanodelivery system of PEG-PCL nanoparticles with encapsulated nucleic acids–mannosylated PEI (Man-PEI) complexes was developed for intestinal delivery. We complexed model nucleic acids with Man-PEI at the optimal N/P ratio of 20:1 for in vitro and in vivo analyses. Cells were transfected in vitro and analyzed for gene expression, receptor-mediated uptake, and PEG-PCL nanoparticles’ toxicity. We also evaluated the nucleic acid’s stability in the nanocarrier during formulation, and under simulated gastrointestinal environments or the presence of nucleases. Finally, we assessed the biodistribution for the PEG-PCL nanoparticles with encapsulated complexes and their ability to transfect intestinal cells in vivo. Nucleic acids complexed with Man-PEI were protected from degradation against nucleases. In comparison to the parent compound PEI, Man-PEI transfected the cells with an overall higher potency. Competition assay indicated receptor-mediated endocytosis promoted by mannose receptors. The PEG-PCL nanoparticles with Man-PEI/plasmid complexes indicated minimal cytotoxicity. The nanocarrier successfully protected the complexes in a simulated gastric fluid environment and released them in a simulated intestinal fluid environment, promoted by the presence of lipases. The oral administration of the PEG-PCL nanoparticles with encapsulated Man-PEI/plasmid complexes transfected intestinal cells with the plasmid in vivo, while presenting a time-dependent progression through the intestines. Conclusively, our carrier system can deliver genetic material to the GI tract and actively target mannose receptor overexpressing cells.

## 1. Introduction

Oral delivery of active pharmaceutical ingredients has been the predominately preferred route of administration due to its simplicity and capacity to induce topical and systemic administration while promoting patient compliance [1]. Among the different types of diseases, gastrointestinal diseases present a unique target for oral drug delivery, as it can achieve a direct and topical drug presence in the intestinal tissues without the need for or the side effects associated with systemic absorption or circulation. Inflammatory Bowel Diseases (IBD), such as Crohn’s disease and colitis, as well as intestinal and colorectal cancers (CRC), are prominent examples of gastrointestinal diseases. Briefly, IBD is frequently observed in western countries and it is estimated that approximately 1.3% of adults are diagnosed with either Crohn’s or ulcerative colitis in the US alone [2]. Intestinal and CRC are a leading cause of cancer-related mortality and morbidity worldwide, accounting for the third-highest incidence and death rate in men and women [3]. Despite the recent advent of novel approaches, the current standard of care therapies can have low specificity, which correlates to significant side effects during treatment [4,5], or present disease relapse [6,7].

IBD and intestinal cancers are associated with several gene dysregulations. Nucleic acid-based therapeutics present a promising approach for the treatment and/or prevention of the two aforementioned diseases [8,9]. TNF-a, IFN-γ, IL-4, IL-10 IL-21, activation of proto-oncogenes (KRAS) and inactivation of genes like APC, and p53, are some of the examples of gene dysregulations or genetic alterations during IBD and intestinal cancers [10,11,12,13,14,15]. Nucleic acid-based therapeutics, such as plasmids, small interfering RNAs (siRNAs), microRNAs (miRNAs), and messenger RNAs (mRNAs), are versatile molecules that can regulate gene expressions and disease progression [16].

As patients with IBD have a 4.5-fold increased risk of CRC and both diseases may require protracted therapies [17], the oral route of delivery for nucleic acids presents a potential benefit for long-term treatments against either disease. Unfortunately, the oral delivery of nucleic acids presents significant challenges. The stomach’s acidic environment and the enzyme-rich intestine present significant hurdles for the oral administration of nucleic acids [1,16,18]. Therefore, a system capable of delivering nucleic acids to the intestinal cells following oral administration can provide significant advantages. Furthermore, the development of a delivery carrier that can not only transfect intestinal tissue, but also preferentially target cells of the immune system or cancer cells presents significant advantages over traditional approaches.

Nanotechnology-based approaches have propelled active targeting methodologies against cancer cells [19]. For example, active targeting can be achieved by the direct conjugation of ligands, such as small molecules, proteins and peptides, onto the nanoparticles’ surface to hone towards receptors overexpressed in specific cell types [19]. Representative examples include the Arg-Gly-Asp (RGD) peptide that binds to integrins [20], folic acid that binds to the folate receptors [21], and chlorotoxin that binds to MMP-2 receptors [22]. Here, we report on the development of a novel delivery carrier for the oral delivery of nucleic acids, capable of targeting cells with overexpressed mannose receptors. This includes cells of the immune system, such as macrophages or cancer cells [23,24].

To evaluate the nanocarrier, we use two model nucleic acids, pGL3 luciferase-expressing plasmid and green fluorescence protein (GFP) expressing plasmid. Briefly, we prepared PEG-PCL nanoparticles (NPs) with encapsulated mannosylated PEI (Man-PEI)/plasmid complexes. The Man-PEI/plasmid complexes transfect cells with the nucleic acids while actively targeting cells with overexpressed mannose receptors, as presented here, for minimizing nonspecific targeting to healthy cells. For Man-PEI, we use PEI1800, due to its minimal toxicity and strong transfection efficiency [1,16]. The encapsulation of the Man-PEI/plasmid complexes inside PEG-PCL NPs protects them during their transition through the harsh acidic gastric environment. The PCL’s solid polymeric core remains stable in the stomach’s acidic environment but degrades in the neutral-to-basic intestinal area, aided by lipase enzymes present at the organ [25], releasing the Man-PEI/nucleic acid complexes locally. As Man-PEI was previously studied for other routes of administration with advantageous and promising results [26,27,28], here we present a new application for the successful oral delivery of nucleic acids with the polymer. Thus, such behavior potentiates passive targeting to the small and large intestine and active targeting through the mannose-receptor to mannose-overexpressing cells.

The nanocarrier is designed to overcome the limitations of oral nucleic acid delivery and can be potentially utilized for the treatment of both intestinal diseases, IBD or cancer. In this study, we focus on the potential targeting to CRC cells due to the disease’s severity on survival. We conclusively demonstrate the expression of a non-endogenous gene in the intestinal tissue following the oral administration of the respective plasmid. Our analysis does not only present a visual and quantitative successful transfection following oral delivery using an exogenous gene but also the potential for intestinal and, more importantly, colonic delivery of nucleic acids with active targeting, based on our nanocarrier system.

## 2. Methods

### 2.1. Materials

Cell culture reagents and trypsin were purchased from VWR (Radnor, PA, USA) or Corning Cellgro (Manassas, VA, USA). Fetal Bovine Serum (FBS) was obtained from Atlanta Biologicals. Opti-MEM was purchased from GibcoTM (Life technologies, Carlsbad, CA, USA). mPEG-PCL (5000:20,000) and PEG-PCL conjugated with aminofluorescein were purchased from PolySciTech Inc. (West Lafayette, IN, USA). α-D-mannopyranosylphenyl isothiocyanate (MPITC) was purchased from Carbosynth Ltd. (Berkshire, UK). Simulated gastric fluid, simulated intestinal fluid, lipase were obtained from Fisher (Hampton, NH, USA). Anti-mannose receptor (CD206) antibody (ab64693) was acquired from Abcam (Cambridge, MA, USA), Alexa Fluor 488 goat anti-rabbit IgG (A11008) was purchased from Invitrogen (Carlsbad, CA, USA). ONE-GLO + Tox Luciferase Reporter and cell viability assay kit were from Promega (Madison, WI, USA). GFP quantification kit (ab235672) was purchased from Abcam (Cambridge, MA, USA).

### 2.2. Cell Cultures and Plasmid DNA

Colon cancer cell lines SW480, HCT-15, HT-29, and normal cells HEK-293, HFL1 were obtained from ATCC. We cultured the SW480 cells in L-15 media, HCT-15 and HT-29 cells in RPMI1640 media, HEK-293 in EMEM media, and HFL1 in F-12K media. All media were supplemented with 10% FBS and 1% antibiotics. Cell cultures were maintained at 37 °C, under a 5% CO_2_ environment, with the exception for SW480 that no CO_2_ was supplemented. We used the pGL-3 luciferase-expressing plasmid DNA (Promega, Madison, WI, USA) in vitro and the green-fluorescent-protein (GFP) expressing pLenti-III-mir-GFP plasmid for the in vivo transfection as model nucleic acids (ABM, Richmond, BC, Canada). We extracted and purified the plasmids using the QIAGEN Plasmid Giga Kit (Chatsworth, CA, USA).

### 2.3. Mannose Receptor Expression

To analyze mannose receptors’ relative expression level in colon cancer vs. normal cell lines, we used the SW480, HCT-15, HT-29 colon cancer cell lines, and the HFL1, HEK-293 normal epithelial cell lines. We harvested approximately 10^6^ cells from each cell line and treated them with anti-mannose receptor antibodies at 1:50 dilution. Following incubation and washing, we treated the cells with the secondary antibody, Alexa Flour 488 goat anti-rabbit IgG, at 1:50 dilution. The expression of mannose receptors was detected in 10,000 events of the gated populations using a BD FACSCalibur Flow Cytometer with CellQuest Pro software (BD Biosciences, Franklin Lakes, NJ, USA).

### 2.4. Synthesis of Mannosylated Polyethyleneimine 1800 (Man-PEI)

We utilized a previously published protocol for the synthesis of Man-PEI, with minor modifications [26]. Briefly, we linked mannose to PEI via a phenyl isothiocyanate bridge using mannopyranosylphenyl isothiocyanate as the coupling reagent. We dissolved 25 mg (80 μmol) of α-D-mannopyranosylphenyl isothiocyanate (MPITC) in methanol (5 mL) and added the resulting mixture to a solution of equal methanol volume containing 23.4 mg PEI 1800 (13 μmol) in a drop-wise manner. We allowed the reaction to progress under stirring at room temperature until no MPITC could be detected by thin-layer chromatography (TLC, methanol: chloroform 1:1 *v/v*). We purified the final product through dialysis (MWCO: 1 kDa) in water and freeze-dried it until further use.

The final product, Man-PEI, was analyzed using ^1^H-NMR (CD_3_OD; JEOL Eclipse ECS-400). We perform Fourier Transformed Infrared Spectroscopy (FTIR) spectroscopic analysis from 400 to 4000 cm^−1^ on Man-PEI and its reaction components using a Spectrum Two FTIR spectrometer (PerkinElmer, Waltham, MA, USA). Data were analyzed with PerkinElmer Spectrum Quant software.

### 2.5. Gel Retardation and DNAse Stability Assay

We evaluated the ability of Man-PEI to complex with a plasmid-based on a gel retardation assay, using agarose gel electrophoresis. Briefly, we complexed the Man-PEI at different nitrogen to phosphate (N/P; indicates the ratio between the number of nitrogen atoms present in the Man-PEI to the phosphate atoms in the nucleic acid) ratios and run the samples in a 1% agarose gel with 0.1 μg/mL ethidium bromide, as previously described [29]. The nucleic acid’s signal was visualized under a Chemidoc Touch Imaging (Biorad, Hercules, CA, USA).

To evaluate the ability of Man-PEI to protect nucleic acid degradation in the presence of DNAses, we complexed the pGL-3 plasmid with Man-PEI at different N/P ratios and incubated the samples in the presence of DNase I (2U of DNase/600 ng of plasmid for 30 min at 37 ºC; naked plasmid with DNAses was used as positive control). Following the deactivation of the DNAses using EDTA, the nucleic acids were released from the complexes using 8% polyacrylic acid (PAA) and run in the agarose gel, as described above.

### 2.6. Transfection Assay for Man-PEI/Plasmid Complexes

We incubated SW480 and HCT-15 cells seeded in 96-well Optical-Bottom Plates (Fisher, Hampton, NH, USA) in the presence of 10 μg of pGL-3 plasmid complexed with either Man-PEI or PEI at various N/P ratios (six replicates per sample). After 6 h of incubation at 37 °C, the transfection media in each well was replaced with fresh complete media. Following 24 or 48 h, we determined the luciferase activity and cell survival of the transfected cells using the ONE-GLO + Tox Luciferase Reporter and Cell Viability Assay Kit, according to the manufacturer’s instructions (Promega, Madison, WI, USA). Transfection was calculated by obtaining the ratio of luminescence of cells over cell survival for each well and sample.

### 2.7. Cellular Uptake Study Using Fluorescent Microscopy and Flow cytometer

We conjugated fluorescein-NHS (excitation/emission: 498/517 nm; Lumiprobe, Cockeysville, MD) to Man-PEI and complexed it with pGL-3 plasmid. We investigated the cellular uptake using (a) fluorescence microscopy with an Olympus BX63 microscope, and (b) flow cytometry using a BD FACS Calibur Flow Cytometer along with CellQuest Pro software. Briefly, for fluorescent microscopy, we incubated SW480 and HCT-15 cells with Man-PEI-fluorescein complexed with pGL-3 plasmid at N/P ratio 20:1 in chambered cell culture slides (Falcon, Corning, NY, USA). Following predetermined incubation periods, cells were washed with 1× PBS, fixed with 4% formaldehyde, and covered with 4′,6-diamidino-2-phenylindole (DAPI)-containing mounting media before visualizing under the microscope.

For the flow cytometric analysis, we incubated the SW480 and HCT-15 cells in the presence of Man-PEI-fluorescein/pGL-3 complexes, as described above, for predetermined periods. Subsequently, the cells were washed with 1× PBS, harvested, fixed with 4% formaldehyde and analyzed under a flow cytometer.

### 2.8. Competition Transfection Assay

We seeded SW480 and HCT-15 cells in 96-well optical-bottom plates (Fisher, Hampton, NH, USA) for transfection with Man-PEI or PEI complexed with pGL-3 at N/P ratio 20:1, as above. Some cells were pretreated with free mannose (1.5 mmoles/liter) for an hour prior to transfection. After 48 h, we determined luciferase activity and cell survival of the transfected cells using ONE-Glo + Tox Luciferase Reporter and Cell Viability Assay Kit, according to the manufacturer’s protocol. We calculated transfection by obtaining the ratio of luminescence over the cells’ survival in each well [29].

### 2.9. Formulation and Characterization of PEG-PCL NPs

To prepare the PEG-PCL NPs with encapsulated Man-PEI/plasmid complexes, we used the double-emulsion solvent evaporation technique with minor modifications, as previously described [30]. Based on the transfection data, we complexed the plasmid with Man-PEI at the optimal N/P ratio of 20:1 in 200 μL of Tris-EDTA, which formed the inner aqueous phase. The oil phase consisted of 100 mg PEG-PCL dissolved in 2 mL dichloromethane (DCM) solution. The outer aqueous phase consisted 6 mL of a sodium cholate solution at a concentration of 6 mg/mL. The inner aqueous phase was transferred into the oil phase and emulsified the system using a probe sonicator for 30 s (Misonix Ultrasonic liquid processors, 15 output wattage), to form the primary-water-in-oil (*w/o*) emulsion. We subsequently placed this emulsion in the outer aqueous phase and probe-sonicated for 60 s to form the final water-in-oil-in-water (*w/o/w*) emulsion. We allowed the DCM to evaporate under continuous stirring for approximately 3 h. We collected the NPs via centrifugation at 14,000× *g* for 1 h and washed them using nuclease-free water. The supernatant obtained from the centrifugation was collected to calculate the plasmid’s encapsulation efficiency, using the PicoGreen assay (Invitrogen, Waltham, MA, USA) at wavelengths of 485/520 nm. We determined the plasmid loading in NPs by subtracting the total amount of plasmid recovered in the supernatant from the initial amount of plasmid added into the formulation. We calculated the encapsulation efficiency, loading capacity, and production yield of the NPs according to the following equations [31]:

(1) $$Encapuslation\;efficiency\;\left(\%\right)=\frac{\mathrm{Initial}\;\mathrm{weight}\;\mathrm{of}\;\mathrm{plasmid}\;\mathrm{added}-\mathrm{weight}\;\mathrm{of}\;\mathrm{plasmid}\;\mathrm{recovered}\;\mathrm{in}\;\mathrm{the}\;\mathrm{supernatant}}{\mathrm{Initial}\;\mathrm{weight}\;\mathrm{of}\;\mathrm{plasmid}\;\mathrm{added}}\times 100$$

(2) $$Production\;yield\;\left(\%\right)=\frac{\mathrm{Weight}\;\mathrm{of}\;\mathrm{produced}\;\mathrm{nanoparticles}}{\mathrm{Initial}\;\mathrm{weight}\;\mathrm{of}\;\mathrm{polymer}+\mathrm{Initial}\;\mathrm{weight}\;\mathrm{of}\;\mathrm{plasmid}\;\mathrm{added}}\times 100$$

We determined the particle size, size distribution and zeta potential of the PEG-PCL NPs using NanoBrook 90Plus PALS (Brookhaven, Holtsville, NY, USA) at room temperature. SEM images of the PEG-PCL NPs were obtained under a FEI Quanta 3D FEG FIB/SEM dual beam system interfaced with the EDAX Apollo XL EDS detector. The powder samples of air-dried NPs were fixed on SEM stub with double-sided carbon tape and then coated with a thin layer of Pt to avoid charging effect during imaging and analysis.

We evaluated the plasmid retention from PEG-PCL NPs with encapsulated Man-PEI/pGL-3 complexes at two N/P ratios, 7:1 or 20:1. Briefly, 10 mg of lyophilized NPs were suspended in 1 mL PBS and incubated at 37 °C for 40 min under constant shaking. Then, the suspension was centrifuged for 15 min at 14,000× *g* and any plasmid was detected using the agarose gel electrophoresis described above.

### 2.10. Cell Viability Assay

We used a standard 3-(4, 5-dimethylthiazol-2-yl)-2, 5-diphenyltetrazolium bromide (MTT) assay to determine the PEG-PCL NPs’ cytotoxicity in SW80 and HCT-15 cells, as previously described [29]. Briefly, we treated SW480 and HCT-15 cells with different concentrations of PEG-PCL NPs (15–1000 μg/mL) containing Man-PEI/plasmid complexes in complete media. Following 24, 48, and 72 h incubation and addition of MTT, the absorption of solubilized formazan crystals in acidified SDS at 570/630 nm was acquired using a plate reader (BioTek H1 Synergy Plate Reader, Winooski, VT). Cell viability was determined as a percentage of the negative control (untreated cells).

### 2.11. Plasmid Release and Stability Assay from PEG-PCL NPs

A PEG-PCL NPs suspension containing 50 μg plasmid complexed with Man-PEI in 10 mL of simulated gastric fluid (SGF), simulated intestinal fluid (SIF), and simulated intestinal fluid with lipases (SIF + lipase; 1 mg/mL). At predetermined time points, 0.5, 1, 2, 4, 8, 24, 48, and 72 h, we withdrew 0.5 mL samples from each release media and collected the supernatant after centrifuging the samples for 20 min at 14,000× *g*. To maintain the volume of the release media unchanged, 0.5 mL of fresh release media was supplemented to each tube, respectively. We measured plasmid concentration with the PicoGreen assay kit, as described above. As SGF’s acidity interfered with plasmid quantification, the measurements took place after dilution with NaOH to neutralize SGF’s acidity. The plasmid detection under the different incubation conditions was confirmed with unentrapped, non-complexed, naked plasmids.

Finally, we evaluated the stability of the plasmid complexed with Man-PEI with or without encapsulation in PEG-PCL NPs and incubated in SIF in the presence or absence of lipases. Briefly, for the NPs, we incubated 10 mg of lyophilized NPs suspended in 1 mL of SIF with lipases (1 mg/mL) for 72 h at 37 °C. Subsequently, we centrifuged the sample for 20 min at 14,000× *g*, and supernatants were collected. The collected supernatant was then run directly into the gel under the presence or absence of polyacrylic acid (PAA). Similarly, the non-encapsulated Man-PEI/plasmid complexes were treated under similar conditions.

### 2.12. In Vivo Biodistribution Assay

We performed a biodistribution analysis of the PEG-PCL NPs in C57BL/6J wild-type female mice (4–6 weeks old, Envigo, Indianapolis, IN, USA). The experimental protocol was approved by the Institutional Animal Care and Use Committee (IACUC; Protocol number: 20OCT-GM-01; October 2020) of the University of Louisiana Monroe, based on Office of Laboratory Animal Welfare (OLAW), National Institute of Health (NIH) guidelines, and the Guide for the Care and Use of Laboratory Animals, 8th ed. For our analysis, we used PEG-PCL polymer tagged with fluorescein (Ex: 492 nm, Em: 516 nm). We formulated the NPs with Man-PEI/plasmid complexes, as described above. To minimize the food’s interference, the mice were under an Alfalfa-free diet for at least five days before our analysis. Furthermore, the mice were fasted for 24 h before oral administration. Subsequently, 12.5 mg of the PEG-PCL NPs in 300 μL suspension were given via oral gavage per mouse. Animals were sacrificed at each predetermined time point (0 h- no dose, 1, 2, 4, 8, and 24 h; 2 animals per time point). We collected the stomach, small intestine, cecum, and colon of the mice and obtained fluorescent images using IVIS (Perkin-Elmer, Waltham, MA, USA).

### 2.13. In Vivo Plasmid Transfection

The transfection potential of our system following oral administration was assessed in C57BL/6J wild-type mice. To detect the in vivo transfection of plasmids in the intestine, we used the GFP-expressing plasmid complexed with Man-PEI at N/P ratio 20:1. The complexes were encapsulated in PEG-PCL NPs, as described above, and given orally to mice (*n* = 3 for treatment, *n* = 2 for control) at 100 μg of plasmid/mouse, daily for five days (1×/day). Subsequently, the mice were sacrificed, and the intestinal tissues were harvested. The tissues were opened, cleaned, rolled, and fixed using cold-isopentane. The samples were sectioned at 20 µm thickness using a cryostat (Leica CM3050 S), such that each section included the complete length of the intestine. Sectioned intestinal tissues were viewed under a fluorescence microscope for GFP signal. For the negative control, mice that were not fed with PEG-PCL NPs were used. As a positive control, we transfected SW480 cells with GFP-expressing plasmid and lipofectamine 2000 for 48 h.

GFP expression in the tissues was verified using GFP quantification kit (Abcam, Cambridge, MA, USA). Briefly, 50 mg of intestinal tissue samples were harvested from untreated and treated mice (two tissue samples per animal, two animals for control, three animals for treatment). The harvested tissues were lysed in 500 μL of GFP assay buffer, using a homogenizer for 60 s. The samples were then incubated in ice for 10 min to ensure complete lysis and centrifuged for 10 min at 10,000 RCF to separate the supernatant. An amount of 100 μL of the supernatant were used to quantify the amount of GF-protein present, following the manufacturer’s protocol.

## 3. Results

### 3.1. Mannose Receptor Expression

**Figure 1:**
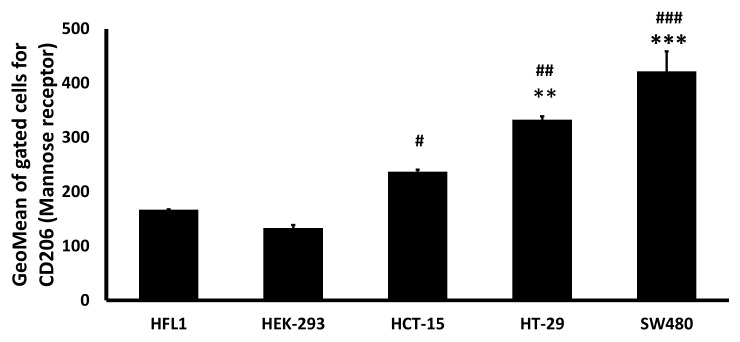
Comparative expression of Mannose receptors between colon cancer and normal epithelial cell lines. Mannose receptor expression on CRC cell lines was higher than the normal epithelial human embryonic kidney cell line (HEK-293) and normal lung fibroblast cell line (HFL1), as detected through flow cytometry. *** *p* < 0.001; ** *p* < 0.01 when compared to HFL1; ### *p* < 0.001; ## *p* < 0.01; # *p* < 0.05 when compared to HEK-293. One-way ANOVA followed by Tukey’s test.

We evaluated the mannose receptor expression in colon cancer cell lines and compared it to normal cell lines. We used three colon cancer cell lines, SW480, HCT-15, HT-29, and two normal cell lines, HEK-293 and HFL1. The expression of the mannose receptor is determined based on the geometrical mean of 10,000 gated cells (two replicates per cell line). The two normal cell lines had a lower expression for mannose receptors compared to all colon cancer cell lines (Figure 1). Specifically, HCT-15 presented higher mannose receptor expression when compared to HEK-293 (*p* < 0.05) and HFL1, though it did not reach statistical significance for the latter. In contrast, HT-29 (*p* < 0.01) colon cancer cells and SW480 (*p* < 0.001) expressed mannose receptors significantly higher compared to either HEK-293 or HFL1.

### 3.2. Synthesis and Characterization of Man-PEI

**Figure 2:**
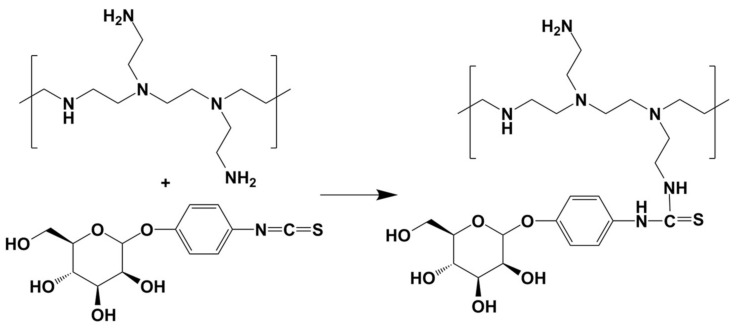
Reaction scheme for the synthesis of Man-PEI. PEI1800 reacted with α-D-Mannopyranosylphenyl isothiocyanate for 24 h to produce Mannosylated PEI. The product was purified through dialysis.

We prepared Man-PEI using α-D-mannopyranosylphenyl isothiocyanate (MPITC) and PEI1800. The synthetic scheme is presented in Figure 2. The MPITC’s ^1^H NMR spectrum contains a peak at nearly 7.2 ppm, which is attributed to its phenyl group. Similarly, the PEI1800′s spectrum has multiple peaks from 2.4 to 2.8 ppm, which are attributed to the –NCH_2_CH_2_– groups [26]. Man-PEI’s spectrum has similar distinct peaks, observed at 2.3–3 and 7.3 ppm. The Man-PEI’s peak integration indicated a MPITC:PEI molar ratio of approximately 4:1, with minimal variation between synthesis batches, which presents a Man-PEI average molecular weight of 3050 (Supplementary Figure S1a) [26].

TLC analysis confirmed the reaction of the two products within 24 h (Supplementary Figure S1b). FTIR analysis of MPITC, PEI1800, Man-PEI and the physical mixture of MPITC and PEI is presented in Supplementary Figure S1c. For MPITC, we detected a peak at 2125 cm^−1^ attributed to –N=C=S, whereas the peak at 1502 cm^−1^ is characteristic of C=C stretching vibration of the phenyl group. For PEI 1800, two peaks at 3281 and 1576 cm^−1^ are attributed to the stretching and bending vibrations of the N–H and–NH_2_ groups, respectively [26]. In comparison, Man-PEI’s spectrum did not present the MPITC’s characteristic band at 2125 cm^−1^, which indicates that the –N=C=S group reacted with the PEI, and a new peak at 1400 cm^−1^ is attributed to the phenyl group, supporting that mannose was linked to PEI [26].

### 3.3. Man-PEI Complexes with Nucleic Acids and Protects Them from Nuclease Activity

**Figure 3:**
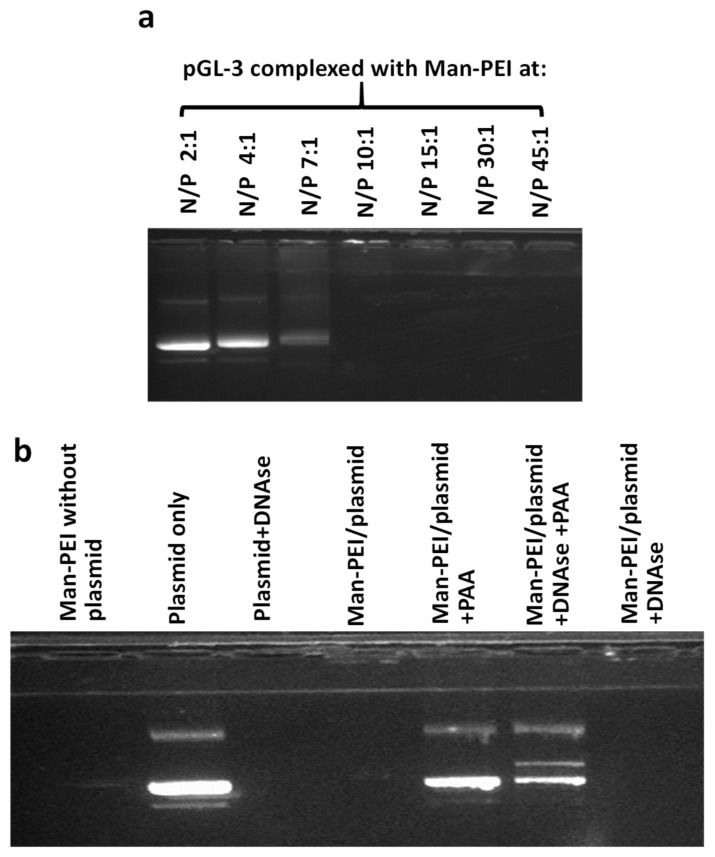
Gel retardation assays through agarose electrophoresis to evaluate Man-PEI’s complexation with plasmid DNA, at different N/P ratios. (**a**) Increasing N/P ratios between Man-PEI and pGL-3 plasmid presented a progressive decrease in plasmid detection in the gel; (**b**) Plasmid alone or complexed with Man-PEI at N/P ratio of 20:1 was incubated in the presence of DNAses I.

We evaluated the complexation of Man-PEI with a model nucleic acid, the pGL-3 luciferase-expressing plasmid, using gel retardation assay at different N/P ratios. At the N/P ratio of 10:1 and above, Man-PEI strongly complexes with the nucleic acids and prevents the migration of the plasmid through the agarose gel (Figure 3a). Treatment with polyacrylic acid (PAA) released the plasmid from the complexes with Man-PEI. Following the incubation of the complexes in the presence of DNAse I nucleases for 30 min at 37 °C, Man-PEI prevented the plasmid’s degradation, as determined following deactivation of the DNAses with EDTA and plasmid release with PAA treatment. As a control, we incubated naked plasmid with DNases, and we did not detect a band (Figure 3b).

### 3.4. Man-PEI/Plasmid Complexes Transfect Colon Cancer Cells

**Figure 4:**
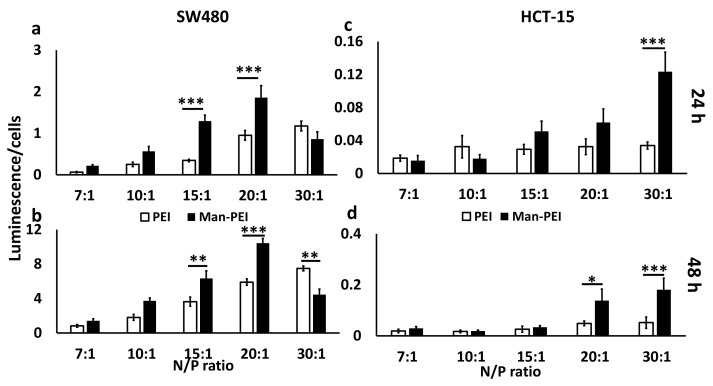
Expression of luciferase as detected by its bioluminescence following transfection of SW480 and HCT-15 cells with pGL-3 luciferase-expressing plasmid complexed with Man-PEI after 24 and 48 h. SW480 (**a**,**b**) and HCT-15 (**c**,**d**) cells were transfected with Man-PEI/pGL-3 complexes and luciferase expression was detected at for 24 (**a**,**c**) and 48 h (**b**,**d**); “Luminescence/% cells” indicates bioluminescence intensity over the number of live cells, as described in Methods section. *** *p* < 0.001; ** *p* < 0.01; * *p* < 0.05 between (□) PEI and (■) Man-PEI groups.

We transfected two cell lines, SW480 and HCT-15, with pGL-3 luciferase-expressing plasmid complexed with Man-PEI at different N/P ratios, spanning from 7:1 to 30:1. The parent PEI molecule was also used for comparison at the same N/P ratios. Transfection was analyzed based on the ratio of luminescence over cell viability (Figure 4). For SW480, Man-PEI induced stronger transfection for N/P ratios between 7:1 to 20:1, when compared to PEI, with the difference reaching statistical significance for N/P ratios of 15:1 (3.7-fold increase at 24 h, *p* < 0.001; 1.75-fold increase at 48 h, *p* < 0.01) and 20:1 (2-fold increase at 24 h, *p* < 0.001; 1.76-fold increase at 48 h, *p* < 0.001). In contrast, PEI induced stronger transfection at the N/P ratio of 30:1, though only significant at 48 h (1.67-fold increase, *p* < 0.01). For HCT-15, Man-PEI induced a stronger transfection compared to PEI at all N/P ratios and time points, though the differences reached statistical significance at N/P ratio of 30:1 at 24 h (4-fold increase, *p* < 0.001), and 20:1 (3.3-fold increase, *p* < 0.05) and 30:1 (3.6-fold increase, *p* < 0.001) at 48 h. Based on these results, we chose as optimal N/P ratio the 20:1, which we used for any subsequent analyses.

### 3.5. Cell Uptake of Man-PEI/Plasmid Complexes Is Promoted by Receptor-Mediated Endocytosis

**Figure 5:**
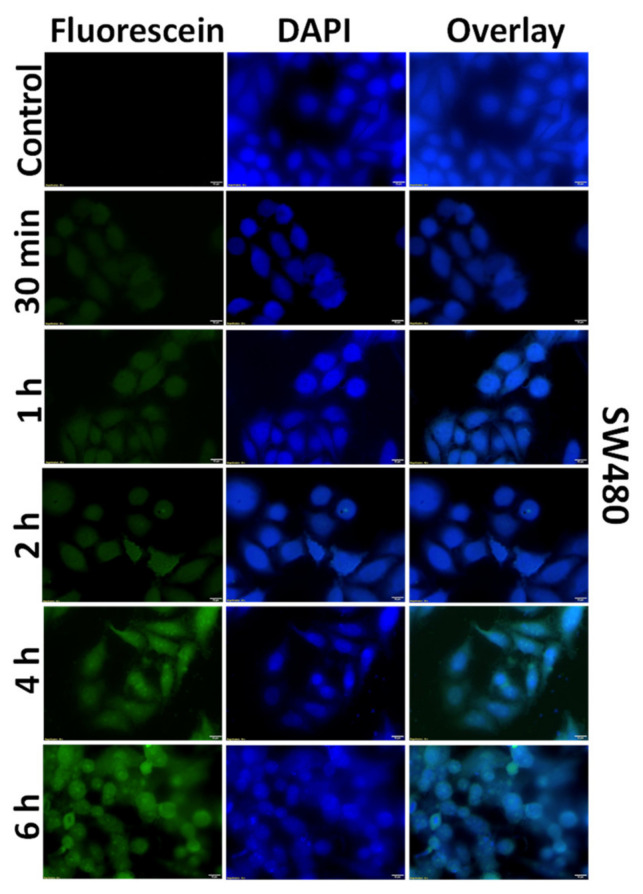
Cellular uptake of Fluorescein-labelled Man-PEI/plasmid complexes by SW480 cells. Incubation of Man-PEI-fluorescein/plasmid complexes with SW480 indicated a time-depended increase in the cellular uptake, as observed by fluorescence microscopy.

**Figure 6:**
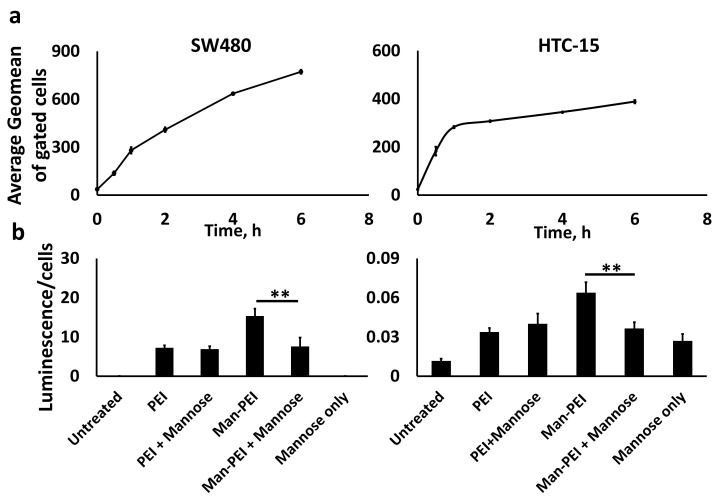
Flow cytometric analysis for detecting Man-PEI/plasmid complexes’ cellular uptake and competitive transfection assay. (**a**) Man-PEI-Fluorescein/plasmid complexes presented a time-dependent uptake in SW480 and HCT-15, as detected by flow cytometry; (**b**) Competition transfection analysis of Man-PEI/plasmid complexes compared to PEI/plasmid complexes. Both polymers were complexed with pGL-3 luciferase-expressing plasmid at N/P ratio 20:1. Our analysis presents the luciferase expression over cell viability for each respective sample and time point, as described in the Methods section; “+Mannose” indicates a 1-h pretreatment of the cells with free mannose prior to transfection. ** *p* < 0.01. One-way ANOVA with post-hoc Tukey’s test.

We used the fluorescein-tagged Man-PEI complexes to investigate the internalization of the complexes into two colon cancer cell lines, SW480 and HCT-15. Both cell lines were treated with the tagged polymer for different predetermined time periods, i.e., 30 min, 1, 2, 4, 6 h, and analyzed by either fluorescent microscopy or flow cytometry. As shown in Figure 5 for SW480 and Supplementary Figure S2 for HCT-15, we observed a time-dependent increase of the Man-PEI/plasmid complexes’ uptake in both cell lines. Fluorescein and DAPI channels were used to detect the complexes and the cell nuclei, respectively, using fluorescent microscopy. These results were quantitatively corroborated using flow cytometry (Figure 6a).

To confirm the receptor-mediated endocytosis, we performed a competition transfection assay with SW480 and HCT-15. The cells were transfected with either Man-PEI or PEI complexes with pGL-3 for 48 h, and some of the wells received 1 h incubation with free mannose prior to transfection. As shown in Figure 6b, mannose pretreatment of the cells significantly reduced the transfection capacity of Man-PEI by 51% in SW480 (*p* < 0.01) and by 42% in HCT-15 (*p* < 0.01), when compared to the cells without the mannose pretreatment. Interestingly, mannose pretreatment reduced Man-PEI transfection capacity to approximately the same levels as its parent compound, PEI (no significant differences between the two groups). Furthermore, mannose pretreatment did not affect PEI’s transfection capacity. These results suggest that Man-PEI uptake is promoted via a mannose receptor-mediated endocytic process.

### 3.6. Characterization of PEG-PCL NPs

**Figure 7:**
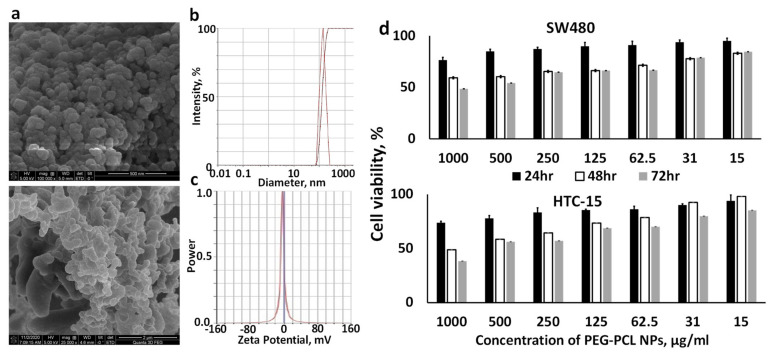
PEG-PCL NPs with encapsulated Man-PEI/plasmid complexes present nanosized spherical structures of relatively similar size and minimal toxicity. (**a**) Representative SEM imaging of the polymer nanocarriers; (**b**) Dynamic Light Scattering analysis of the nanoparticles; (**c**) Zeta-potential analysis of the nanocarriers; (**d**) Cytotoxicity analysis of PEG-PCL NPs with encapsulated Man-PEI/plasmid complexes in SW480 and HCT-15 cells.

PEG-PCL NPs with encapsulated Man-PEI/plasmid complexes were prepared using the double emulsion/solvent evaporation method and characterized for morphology, size, and zeta potential, using SEM, DLS, and microelectrophoresis, respectively. The NPs were spherical, as seen in the SEM, and with an average size of 142.95 nm ± 4.06, a PDI of 0.094 ± 0.031, and a zeta potential of 5.6 mV ± 0.9. The encapsulation efficiency of pGL-3 in PEG-PCL NPs was 78.26% ± 2.12 (Figure 7 and Table 1).

### 3.7. PEG-PCL NPs Have Minimal Effect on Cell Viability

We evaluated the cytotoxicity of PEG-PCL NPs with encapsulated Man-PEI/pGL-3 plasmid complexes using MTT by incubating them at different concentrations with SW480 and HCT-15 cells for 24, 48 and 72 h, which indicated overall minimal toxicity by the NPs. Briefly, the NPs’ concentrations ranged between 15 and 1000 μg/mL. For SW480 cells, the 24 and 48 h treatments did not present an IC50 value, while for 72 h, the IC50 value was 819 μg/mL. For HCT-15, the 24 h treatment did not present an IC50 value at the studied concentrations. However, the IC50 value was 831 and 651 μg/mL at 48 and 72 h, respectively (Figure 7d).

### 3.8. Plasmid Release from PEG-PCL NPs and Stability In Vitro

**Figure 8:**
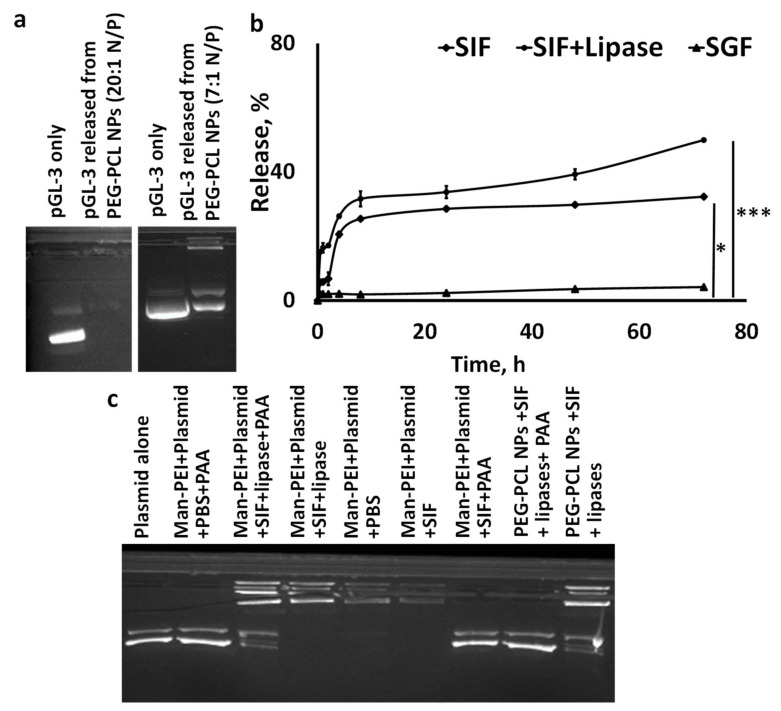
Plasmid release and plasmid stability studies, using PEG-PCL NPs with encapsulated Man-PEI/plasmid complexes, under simulated gastrointestinal conditions. (**a**) Plasmid retention in PEG-PCL NPs with encapsulated Man-PEI/plasmid complexes at N/P ratio of 20:1 vs. 7:1; (**b**) Amount of plasmid released from the PEG-PCL NPs with encapsulated Man-PEI/plasmid complexes, as a function of time, under SGF and SIF. The latter was evaluated in the presence or absence of lipases. * *p* < 0.05; *** *p* < 0.001; (**c**) Gel electrophoresis analysis of the plasmid released from either Man-PEI/plasmid complexes (Man-PEI+plasmid; Lanes 2–7) or from PEG-PCL NPs with encapsulated Man-PEI/plasmid complexes (Lanes 8 and 9), in the presence and absence of lipases. A comparison between PBS and SIF also takes place.

We evaluated the plasmid retention from PEG-PCL NPs encapsulating Man-PEI/p-GL-3 complexes at two N/P ratios, to ensure that strong complexation between Man-PEI/plasmid retains the plasmid within the PEG-PCL NPs. For the lower N/P ratio of 7:1, the plasmids were detected in the agarose gel. However, for N/P ratio of 20:1, the plasmid band was not detected, indicating the strong complexation between the plasmids and Man-PEI while encapsulated in the PEG-PCL NPs (Figure 8a).

We evaluated the in vitro release of pGL-3 from PEG-PCL NPs vs. time, presented as a percentage of plasmid release in Figure 8b. The study took place with three release media; SGF, SIF, and SIF+lipase. There was minimal plasmid release in the SGF up to 72 h. In contrast, we detected a significantly higher plasmid release for the nanoparticles incubated in SIF compared to SGF (*p* < 0.05), where approximately 32% of the plasmid was released from the matrix at 72 h. Upon addition of lipase (1 mg/mL) to the system, plasmid release from the nanoparticles was accelerated and increased to approximately 50% in 72 h. Lipases promote PCL degradation [25].

We performed agarose gel electrophoresis to evaluate the plasmid stability in the NPs under various conditions. In Figure 8c, we detect under different conditions the plasmid released from: (a) the non-encapsulated Man-PEI/plasmid complexes in lanes 2–7, and (b) the Man-PEI/plasmid complexes encapsulated in PEG-PCL NPs in lanes 8 and 9. Briefly, plasmid detection from Man-PEI/plasmid complexes was only feasible following incubation of the complexes with PAA, while SIF (with or without lipases) did not release nor degrade the plasmid (Lanes 2–7). As we showed in Figure 8a, the PEG-PCL NPs with encapsulated Man-PEI/plasmid complexes at N/P ratio of 20:1 were strongly retaining the plasmid. In contrast, released plasmid from the PEG-PCL NPs was detected after incubation with SIF+lipases, but only with co-incubation with PAA (Lanes 8 and 9). In summary, these results indicate that: (a) the Man-PEI/plasmid complexes are stable in SIF and the presence of lipases; (b) the SIF + lipases promote the release of the plasmid/Man-PEI complexes from the PEG-PCL NPs; (c) the encapsulation process does not damage the plasmid, and; (d) the plasmid is released from the PEG-PCL NPs together and still complexed with Man-PEI, as it requires PAA in order to be detected (Lane 9 vs. 8). In summary, these results indicate that the encapsulation of Man-PEI/plasmid complexes inside PEG-PCL NPs prevents the plasmid release and degradation in SGF, which is then released and still protected in the SIF, as it remains complexed with Man-PEI.

### 3.9. In Vivo Biodistribution of Orally Administered PEG-PCL NPs Presented Gradual Progression through the Intestinal Tract

**Figure 9:**
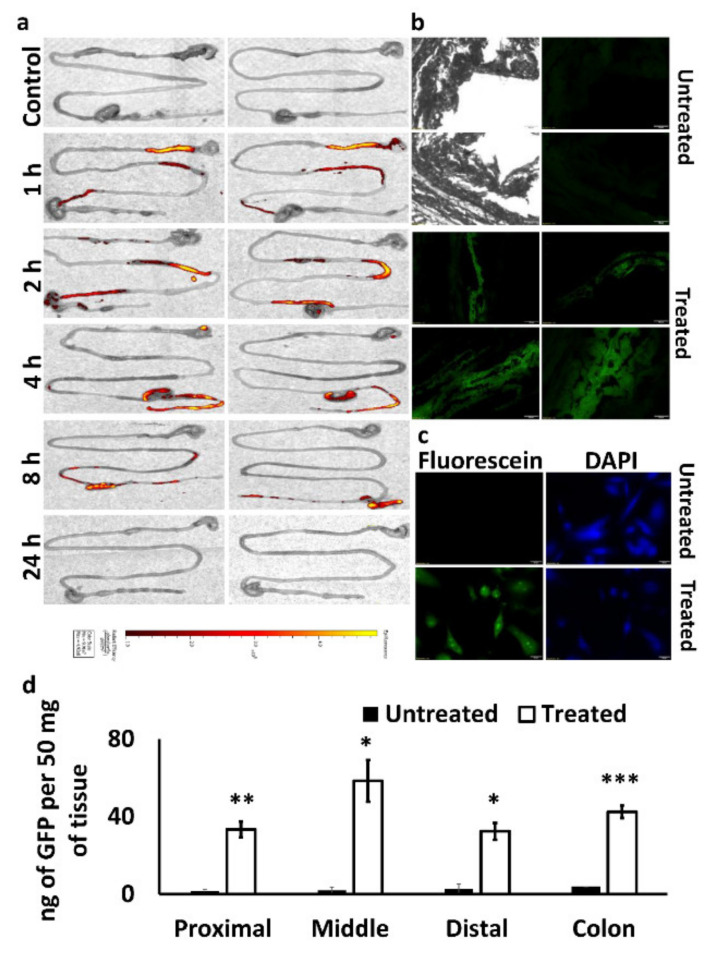
In vivo evaluation of PEG-PCL NPs with encapsulated Man-PEI/plasmid complexes, following oral administration. (**a**) Ex vivo imaging of the biodistribution of fluorescently-labeled PEG-PCL NPs in the intestinal tract of mice (*n* = 2) following their oral administration; (**b**) fluorescence imaging of cryosections from the intestinal tract of mice without any treatment (upper 4 images; the two on the left are bright-field images of the respective spots in the two right Fluorescein images) and mice that were treated with GFP-expressing plasmid complexed with Man-PEI and encapsulated in PEG-PCL NPs; (**c**) SW480 cells were incubated with GFP-expressing plasmid and lipofectamine that served as a positive control for the GFP expression; (**d**) GFP quantification in intestinal tissues obtained from untreated (control mice) and treated (mice treated with GFP-expressing plasmid/Man-PEI complexes in PEG-PCL NPs; see Method section) groups. *** *p* < 0.001 ** *p* < 0.01 * *p* < 0.05 using two-tailed *t*-test.

We evaluated the biodistribution of PEG-PCL NPs with encapsulated Man-PEI complexes in vivo in C57BL/6J wild-type mice. We used fluorescein-labeled PEG-PCL to form NPs and administered them via oral gavage into mice. At predetermined time points, the animals were euthanized, and their intestines were visualized under IVIS. As shown in Figure 9a, 1 h after oral gavage, the NPs were passing through the stomach to the duodenum area. Two hours after the administration, the NPs reached the mid-intestinal region around the jejunum and gradually progressed in the following hours to the ileum, cecum and colon. At the 24-h timepoint, no fluorescent signal was detected, indicating the removal of the NPs from the body or degradation of the dye.

### 3.10. Oral Administration of PEG-PCL NPs with Encapsulated Man-PEI/Plasmid Complexes Induced Protein Expression of the Exogenous Gene in the Intestinal Tissue

The transfection potential of PEG-PCL NPs with encapsulated Man-PEI complexed with a GFP-expressing plasmid upon oral administration was visualized under fluorescence microscopy. We administered the NP formulation to C57BL/6J wild-type mice daily at 100 μg of plasmid/mouse. After 5 days of administration, the animals were euthanized, and intestinal tissue cryosections were visualized for detection of the green fluorescent protein under a fluorescence microscope. As shown in Figure 9b, GFP signal was detected in the intestinal walls, indicating that the GFP plasmid reached the intestines and was transfected into the cells. The negative control samples (tissue from animals that were not treated with the formulation) did not produce a similar signal. As a positive control for GFP expression due to the plasmid, we used SW480 cells transfected with the GFP-expressing plasmid and lipofectamine 2000. Similarly, GFP expression in the intestinal tissues analyzed via a GFP quantification kit showed that the NPs-fed mice presented significantly higher (*p* < 0.05) GFP expression compared to untreated animals at different parts of the intestinal tracts.

In comparison to the respective sections from the untreated mice, the treated mice had (50 mg of intestinal tissue per mouse per group): (a) 33 ng vs. 1.6 ng of GFP protein (Proximal); (b) 58.4 ng vs. 1.9 ng of GFP protein (Middle); (c) 32.4 ng vs. 2.8 ng of GFP protein (Distal), and (d) 42.5 ng vs. 3.8 ng of GFP (colon). (Figure 9d). These data indicate that the plasmid transfection due to the 5-day oral administration of the PEG-PCL NPs induces relatively homogenous gene expression to the intestinal tissues, with the strongest expressions taking place at the middle portion of the small intestine and the colon.

## 4. Discussion

Nanoparticles incorporating nucleic acids have primarily focused on the intravenous route of administration, but the process is invasive and unsuitable for prolonged therapy [32]. In contrast, oral administration has multiple advantages, such as sustained and controllable delivery, ease of administration, patient compliance, and efficiency in targeting diseases of the gastrointestinal tract, such as IBD or colon cancer [1,33]. Oral administration of nucleic acids presents challenges, such as the harsh acidic condition in the stomach with pH 1–2.5, gastric enzymes like pepsin, strong nuclease activity, and the presence of a mucus layer that is difficult to surpass [32,33]. The development of a carrier system that could overcome these barriers and provide an efficient nucleic acid delivery will propel significant advances in nucleic acid-based therapies against diseases related to the gastrointestinal tract through oral delivery.

In this study, we formulated a polymer-based nanocarrier system encapsulating nucleic acids, to overcome the above-presented barriers, when given orally and transfect the nucleic acids to the intestinal tissue. We selected for our study polyethylene glycol (PEG) and poly-caprolactone (PCL) polymers that are FDA-approved, biocompatible, biodegradable, and non-immunogenic [34,35]. PEG, a neutral, hydrophilic polymer, has been widely used in drug delivery applications to endow nanocarriers with long-circulating properties, protect them from trypsinization and increase their hydrophilicity [36]. Furthermore, pegylated nanoparticles transverse through the mucus layer more efficiently than non-pegylated or larger (micro-sized) particles [1]. PCL permits slow drug release and does not get degraded in the acidic gastric pH. However, the PCL matrix gets degraded in the intestinal tissue under the increasingly basic environment, accelerated by the presence of lipases, which are abundantly present in the intestinal tract [37]. Polyethyleneimine (PEI) is a widely used cationic polymer for cell transfection with nucleic acids. PEI demonstrates a buffering capacity over a wide pH range, and it prevents hydrolysis in endosomes. This helps the PEI–nucleic acid complex escape from the endosomal compartment through a proton sponge mechanism [16,29,38]. 

This study uses PEI conjugated with mannose (Man-PEI) as a transfecting agent. Mannose receptors, 175-kDa transmembrane proteins of the C-type lectin family, are overexpressed in the different cells, including cells of the immune system [39] or colon cancer cells [40]. In the interest of focusing our research, and the higher impact of CRC on patient survival, we focus on CRC in this study. Here, we confirmed mannose receptor overexpression in colon cancer cells vs. normal epithelial cells using flow cytometry, which indicated an up to a 3.1-fold increase of mannose receptor expression in cancer cells compared to the normal epithelial cells.

Following the Man-PEI’s synthesis and characterization via FTIR and NMR, we confirmed its ability to complex with the negatively charged plasmid DNA and protect it from degradation by DNAse I, using agarose gel electrophoresis. Although at lower N/P values, i.e., 2:1, 4:1, 7:1, Man-PEI did not efficiently complex with the plasmids, at N/P ratios of 10:1 and above, Man-PEI strongly complexed with the plasmid, and protected it from DNAse-mediated degradation. Our study aligns with previous findings [29,41].

We evaluated Man-PEI’s transfection capacity in SW480 and HCT-15 cells at different N/P ratios and compared it to PEI. As the N/P ratio increased, the luciferase expression, due to the transfection of the pGL-3 luciferase-expressing plasmid, becomes stronger for both polymers, as detected by luminescence, though the Man-PEI induced an overall stronger luciferase expression when compared to PEI for the same N/P ratios. Representatively, in SW480, at the N/P ratio of 20:1, Man-PEI had approximately a two-fold and 1.76-fold higher transfection compared to PEI, at 24 and 48 h, respectively. In contrast, PEI induced stronger transfection at the N/P ratio of 30:1, though only significant at 48 h. For HCT-15, Man-PEI induced stronger transfection compared to PEI at all N/P ratios and time points, though the differences reached statistical significance at N/P ratios of 30:1 at 24 h, and 20:1 and 30:1 at 48 h. These results indicate that mannosylation of PEI is overall increasing the transfection potential of the polymer.

The critical role of a drug delivery system is the successful uptake of its load by the cells. Our analysis confirmed a time-dependent uptake of the Man-PEI/plasmid complexes by the cells using a fluorescence microscope and flow cytometer. As mannose receptors undergo high-affinity binding to agents containing mannose residues, triggering their transport into endocytic pathways [42], we evaluated whether Man-PEI can promote a receptor-ligand binding and endocytosis. Through a competition transfection assay using SW480 and HCT-15 cells that were preincubated with or without free mannose, we determined that pretreatment of cells with free mannose significantly decreased the luminescence compared to cells without mannose pretreatment, i.e., luciferase activity produced from successful transfection with the pGL-3 luciferase-expressing plasmid, after 48 h. In comparison, we detected no significant difference in the control groups, i.e., transfection with PEI (non-mannosylated) with or without pretreatment with free mannose. The purpose of pretreating the cells with free mannose is to saturate the mannose receptors beforehand. Doing this creates a competitive environment between free mannose and mannose present in Man-PEI to bind to mannose receptors. Thus, the mannose receptors’ availability plays a significant role in the enhanced uptake of the Man-PEI/plasmid complexes and they promote the internalization of the mannosylated complexes via a mannose receptor-dependent mechanism. The observed difference in the transfection ability between the mannosylated and parent PEI described above is also supported by the fact that mannose receptor-mediated endocytosis has previously been studied and the receptor has been characterized as a rapid and highly effective endocytic receptor [24,43,44].

The major concern for oral delivery of nucleic acids is to protect them from the harsh gastric environment. To protect the Man-PEI/plasmid complexes, we encapsulated them inside PEG-PCL NPs. Although oral administration does not inherently present limitations in particle sizes, it has been reported that particles ranging between 200 and 500 nm can efficiently transport through the mucus layer [45]. As detected by DLS and SEM analysis, the PEG-PCL NPs possessed an ~140 nm diameter and were spherical.

We evaluated the release of plasmid from PEG-PCL NPs using different simulated gastrointestinal conditions. The incubation of the NPs in a simulated gastric fluid (SGF) induced a minimal release of the plasmids up to 72 h. In contrast, incubation of the NPs in SIF presented an increased plasmid release, further accelerated by the presence of lipases. More importantly, through agarose gel electrophoresis, we determined that the plasmid release from the PEG-PCL NPs takes place with the plasmid still complexed with Man-PEI. This is important, as the purpose of the PEG-PCL is to protect the Man-PEI/plasmid complexes from the gastric environment, but release them in the intestinal environment. At that point, the Man-PEI will protect the plasmids from any nuclease activity, preferentially targeting cells with overexpressed mannose receptor and ensuring successful cell transfection. It is important to note that although a small amount of plasmid was released from the PEG-PCL NPs without being complexed with Man-PEI, the majority of the plasmid maintained its complexation with Man-PEI.

We evaluated in vivo the PEG-PCL NPs biodistribution and their ability to transfect the intestinal area. Following oral administration of fluorescein-labeled PEG-PCL NPs with encapsulated Man-PEI/plasmid complexes and in vivo imaging of the excised stomach, small intestine, and colon tissue, we detected a gradual progression of the fluorescence signal from the stomach area to the colon as a function of time. The fluorescence signal reached the cecum within 2 h of administration and progressed to the colon within 8 h. 24 h post-administration, no fluorescence signal was detected. Although the fluorescein was attached to the PEG-PCL through an ether-triazine type bond, which should minimize leaching of the tag out the NPs, it is not possible to confirm if the observed signal originates from intact, partially broken, or entirely degraded NPs and freed fluorescein. Thus, the signal observed during the later time points may originate from a mixture of the above. A more reliable evaluation of the system is its capacity to transfect the intestinal tissue, as a successful expression of exogenous genes can only be achieved if the system successfully transfects cells.

We used a model plasmid, which induced the expression of the GFP protein and evaluated the intestinal tissue under fluorescence microscopy, following oral delivery. The GFP-expressing plasmid was complexed with the Man-PEI and the complexes were entrapped in PEG-PCL NPs. After five days of daily administration of the NPs, we harvested the intestinal tissue and observed them under the microscope. The green fluorescence seen in the mice’s intestinal tissue sections confirmed the successful transfection of the intestinal tissue. The GFP expression was also confirmed through GFP quantification, indicating a strong expression of the protein along the intestinal tissue. More importantly, the GFP expression in the colon remained high and comparable to the prior intestinal sections. Although the colon presented a relatively higher GFP expression than the proximal or distal portions of the small intestines, the GFP was overall evenly expressed along the intestine. Nonetheless, this indicates that the transfection was feasible throughout the intestinal tissues and up to the colon tissue; thus, the proposed system can efficiently reach the lower section of the intestinal tissue, which is also necessary for colon cancer treatments.

Overall, this behavior indicates that the proposed PEG-PCL NPs can effectively orally deliver nucleic acids to the intestines and transfect the intestinal tissue for the potential treatment of IBD or CRC. This study provides compelling evidence that the formulation can protect nucleic acids from stomach acids, release the Man-PEI/nucleic acid complexes in the intestinal tract through PEG-PCL’s degradation in the tissue, and actively target a mannose receptor-overexpressing cells through the mannose’s decoration of the PEI.

## 5. Conclusions

We developed a novel nanocarrier for overcoming the hurdles present in the oral administration of nucleic acids for its potential utilization in IBD and intestinal cancer treatments. We successfully formulated PEG-PCL NPs encapsulating Man-PEI/plasmid complexes. The complexes transfected cells and presented receptor-mediated endocytosis via the mannose receptors. In vivo, the orally administered NPs gradually progressed through the intestines to the colon and induced GFP expression, following successful transfection. Thus, this nanocarrier merits further evaluation in therapeutic modalities using nucleic acid-based treatments through oral administration.

## 6. Statistical Analysis

The experimental data obtained were analyzed using GraphPad Prism 5 software. Statistical analysis was performed using one-way ANOVA, followed by post-hoc Tukey’s Test, to determine any significant differences among the groups, unless otherwise stated. All data are presented as mean values ± standard errors; *p*-value < 0.05 was considered statistically significant.

## Figures and Tables

**Table 1:** Nanoparticle characterization.

Formulation	Average Hydrodynamic Diameter, nm ± SD	Average Polydispersity Index ± SD	Average Zeta Potential, mV ± SD	Encapsulation Efficiency, %	Yield, %
PEG(5k)-PCL(20k) NPs with Man-PEI(1.8k) and plasmid	142.95 ± 4.06	0.094 ± 0.031	−5.6 ± 0.9	78.26 ± 2.12	70 ± 3.36

## Data Availability

Not applicable.

## Supplementary Materials

The following are available online at https://www.mdpi.com/article/10.3390/pharmaceutics13071075/s1, Supplementary Figure S1: Characterization of Man-PEI (a) NMR spectrum of PEI, MPITC and PEI-Mannose; (b) FT-IR of PEI-Mannose and its components and their physical mixture; (c) TLC of Mannose, PEI-1800 and the PEI-Mannose product. Supplementary Figure S2: Cellular uptake of Fluorescein-labeled Man-PEI/plasmid complexes by HCT-15 cells. Incubation of pGL-3 with fluorescently labeled Man-PEI complexes with HCT-15 cells indicated a time-dependent increase in the cellular uptake, as observed by fluorescent microscopy.

## Abbreviations

PEI	Polyethylenimine
Man-PEI	Mannosylated polyethylenimine
PEG	Polyethylene glycol
PCL	Polycaprolactone
GI	Gastrointestinal
CRC	Colorectal cancer
NP	Nanoparticles
MPITC	α-D-mannopyranosylphenyl isothiocyanate
FTIR	Fourier-transform infrared spectroscopy
N/P	Nitrogen/Phosphate
PAA	Polyacrylic acid
SEM	Scanning Electron Microscopy
SGF	Simulated Gastric Fluid
SIF	Simulated Intestinal Fluid
GFP	Green Fluorescent Protein
PDI	Polydispersity index
MTT	3-(4,5-dimethylthiazol-2-yl)-2,5-diphenyltetrazolium bromide
qPCR	Quantitative polymerase chain reaction
NMR	Nuclear Magnetic Resonance
DLS	Dynamic Light Scattering
DCM	Dichloromethane
mRNA	Messenger RNA
miRNA	Micro RNA
siRNA	Small interfering RNA
