# Supplementary material for: Oral Delivery of Nucleic Acids with Passive and Active Targeting to the Intestinal Tissue Using Polymer-Based Nanocarriers
Source: Pharmaceutics. 2021 Jul 13;13(7):1075. doi: 10.3390/pharmaceutics13071075 (PMC8309160; doi:10.3390/pharmaceutics13071075)
Supplement: Supplementary file 1 [file pharmaceutics-13-01075-s001.zip › pharmaceutics-1254659-supplementary.pdf]

pharmaceutics

Supplementary Materials: Oral Delivery of Nucleic Acids with Passive and Active Targeting to the Intestinal Tissue Using Polymer-Based Nanocarriers

Sagun Poudel, Prabhat R. Napit, Karen P. Briski and George Mattheolabakis

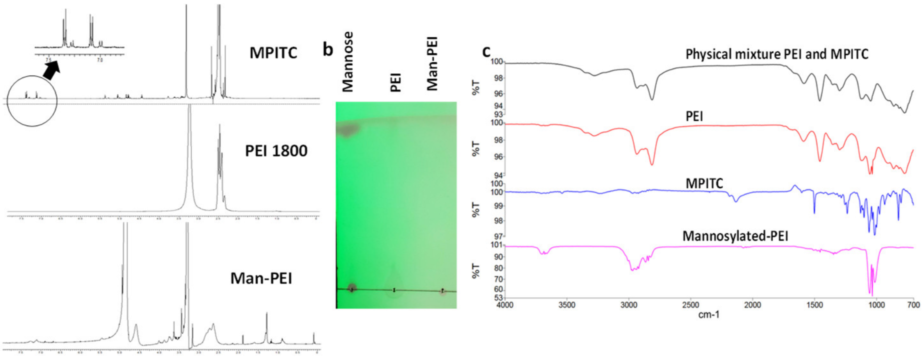

Figure S1. Characterization of Man-PEI. (**a**) NMR spectrum of PEI, MPITC and PEI-Mannose; (**b**) FT-IR of PEI-Mannose and its components and their physical mixture; (**c**) TLC of Mannose, PEI-1800 and the PEI-Mannose product.

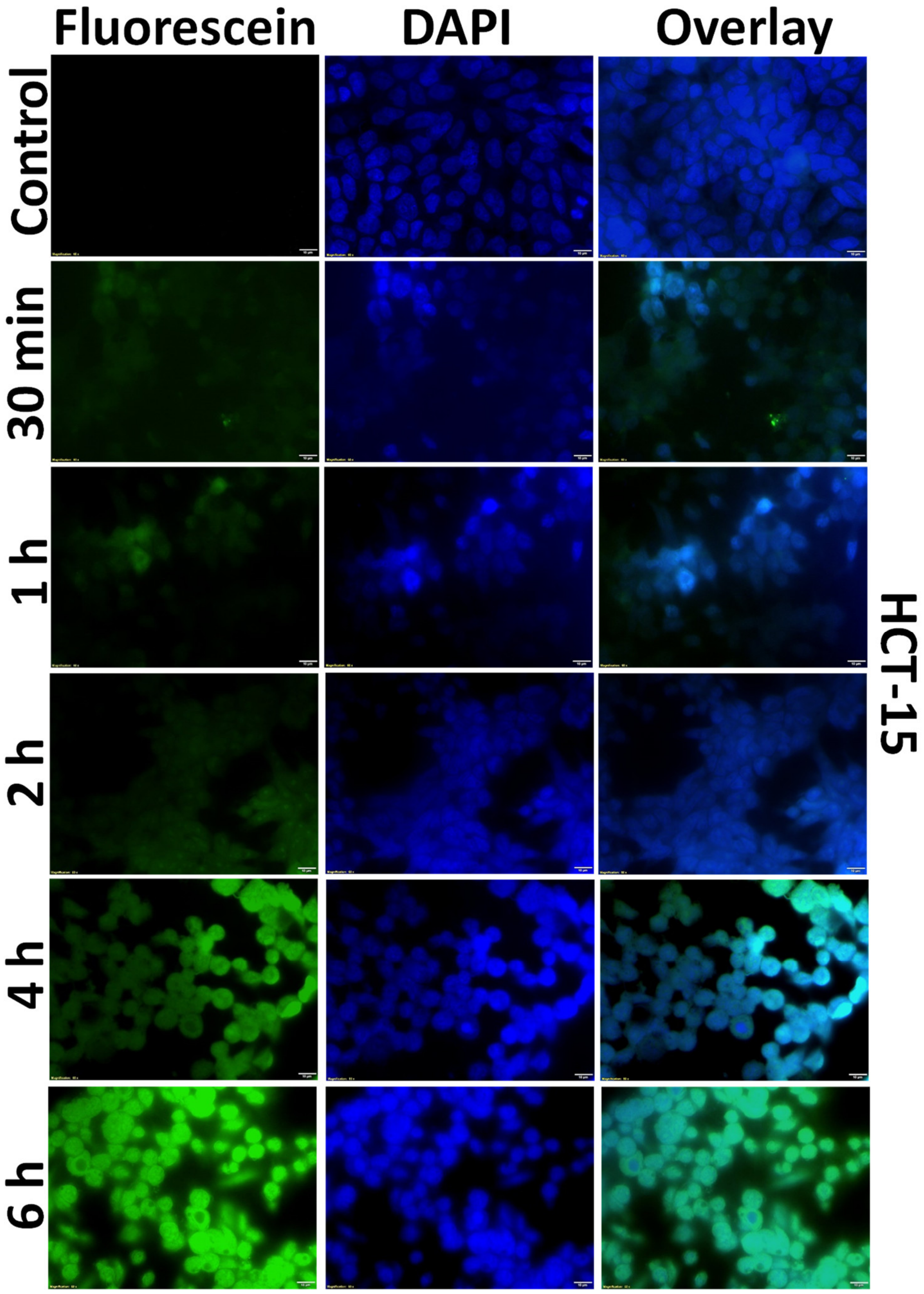

Figure S2. Cellular uptake of Fluorescein-labeled Man-PEI/plasmid complexes by HCT-15 cells. Incubation of pGL-3 with fluorescently labeled Man-PEI complexes with HCT-15 cells indicated a time-depended increase in the cellular uptake, as observed by fluorescent microscopy.
